# A new terrestrial talitrid genus, *Myanmarorchestia*, with two new species from Myanmar (Crustacea, Amphipoda, Talitridae)

**DOI:** 10.3897/zookeys.705.15045

**Published:** 2017-10-02

**Authors:** Zhonge Hou, Shuangyan Zhao

**Affiliations:** 1 Institute of Zoology, Chinese Academy of Sciences, Beijing 100101, China; 2 Southeast Asia Biodiversity Research Institute, Chinese Academy of Sciences, Yezin, Nay Pyi Taw 05282, Myanmar

**Keywords:** taxonomy, *Myanmarorchestia*, COI, Indo-West Pacific, leaf litter

## Abstract

*Myanmarorchestia* Hou, **gen. n.** with two new species is described from terrestrial habitats in Myanmar. This new genus is characterised by 4-dentate lacinia on left mandible, simple gnathopod I in both sexes, weakly chelate gnathopod II in male, simplidactylate pereopods and complex and lobed gills. *Myanmarorchestia peterjaegeri* Hou, **sp. n.** closely resembles *M. seabri* Hou, **sp. n.** in gnathopod II merus and carpus protuberant on posterior margin; however, the former is distinguished from the latter by palp of maxilla I with two articles, coxal gills convoluted, and telson with nicks on surface. Additionally, DNA barcodes of the new species are obtained to confirm their distinctiveness.

## Introduction

The amphipod family Talitridae Rafinesque, 1815 includes two groups, the *Orchestia* group and the *Talitrus* group (Lowry and Coleman 2012). The *Orchestia* group is characterised by well-developed subchelate male second gnathopods and inhabits coastal beach or inland (White et al. 2013). The *Talitrus* group retains the mitten-shaped second gnathopods and occurs in terrestrial habitats (Serejo 2009, Lowry and Coleman 2012). Currently there are 47 genera in the *Orchestia* group (Lowry and Coleman 2012, Wildish and Lecroy 2014), of which four terrestrial genera have been recorded in the Indo-West Pacific margins. The genera *Bousfieldia* Chou & Lee, 1996, and *Lanorchestia* Miyamoto & Morino, 2010 were recorded around Taiwan Island, while *Mizuhorchestia* Morino, 2014 and *Nipponorchestia* Morino & Miyamoto, 2015 were found in Japanese archipelago. The new genus *Myanmarorchestia* with chelate second gnathopods in male displays intermediate shape between *Orchestia* and *Talitrus* groups. Here we assign it to *Orchestia* group, due to its sexually dimorphic gnathopod II. The detailed relationships among these morphological groups need to be assessed with molecular evidences.

For Southeast Asian talitrid fauna, most recorded species were collected near the beach, such as *Talorchestia morinoi* Othman & Azman, 2007 from Tioman Island, Malaysia, *Floresorchestia hanoiensis* Hou & Li, 2003 from Vietnam, and *Floresorchestia samroiyodensis* Azman et al., 2014 from Thailand. Only four species have been found from mountain forest habitats, including *Parorchestia luzonensis* Baker, 1915 and *Curiotalitrus curioi* (Javier & Coleman, 2010) from Philippine Islands, *Parorchestia kinabaluensis* Shoemaker, 1935 from North Borneo, and *Solitroides motokawai* Suzuki et al., 2017 from Vietnam. The genus *Curiotalitrus* with mitten-shaped second gnathopod belongs to *Talitrus* group, while the genera *Parorchestia* and *Solitroides* are uncertain because only female specimens were described.

Mt. Victoria is located in the southwest of Myanmar, known for endemics of montane species (Jäger 2015). In 2014, the arachnologist Dr Peter Jäger of the Senckenberg Research Institute made a field trip to Mt. Victoria to explore the diversity of invertebrates. As a result, eight new species of spiders are found to be vertically niched from 1500 m elevation to the peak (Jäger 2015, Jäger and Minn 2015). Moreover, several specimens belonging to the Talitridae were collected by sieving forest leaf litter from Mt. Victoria, at an elevation of 1585–2150 m. This is the first record of terrestrial Talitridae from Myanmar. Following a detailed examination of the specimens and their genetic data, these amphipods are described as a new genus, *Myanmarorchestia* Hou, gen. n., including two new species *Myanmarorchestia peterjaegeri* Hou, sp. n. and *Myanmarorchestia seabri* Hou, sp. n.

## Materials and methods

### Sampling

The specimens were collected by sieving forest floor litter. Samples were preserved in 95% ethanol in the field, and then deposited at -20°C refrigerator for long preservation. Type specimens are lodged in the Senckenberg Museum, Frankfurt am Main (SMF), Germany, and the Institute of Zoology, Chinese Academy of Sciences (**IZCAS**), Beijing.

### Morphological observation

The body length was recorded by holding the specimen straight and measuring the distance along the dorsal side of the body from the base of the first antenna to the base of the telson. Photos of whole animal were taken with an Olympus C7070 wide zoom digital camera (7.1 megapixels) mounted on an Olympus SZX12 microscope, and they were montaged using Helicon Focus image stacking software. All dissected appendages were mounted on slides in glycerol according to the methods described by Holsinger (1967), and were drawn using a Leica DM2500 compound microscope equipped with a drawing tube. Terminology and taxonomic descriptions follow Morino (2014). The holotype specimen was used for morphological observation, while one paratype specimen was used for both morphological and molecular parts.

### DNA sequencing and COI genetic distance calculations

A partial fragment of the mitochondrial cytochrome oxidase subunite I (COI) was proposed as a crustacean barcode (Costa et al. 2007, Hou et al. 2009). The primers used are CRUSTF2 (5'-GGTTCTTCTCCACCAACCACAARGAYATHGG-3') and HCO2198 (5'-TAAACTTCAGGGTGACCAAAAAATCA-3'). Genomic DNA extraction, amplification, and sequencing procedures were performed as in Hou et al. (2007). All sequences were deposited in GenBank.

The COI sequences were manually aligned, because no indels were observed. Pairwise comparison of uncorrected *p*-distances for two COI sequences obtained in this study was calculated using MEGA7.0.16 (Kumar et al. 2016).

## Taxonomy

### Family Talitridae Rafinesque, 1815

#### Genus Myanmarorchestia

Taxon classificationAnimaliaAmphipodaTalitridae

Hou gen. n.

http://zoobank.org/4EF3E7E6-3D4C-40A3-B294-3E79DB0D2EE2

##### Type species.

*Myanmarorchestia peterjaegeri* Hou, sp. n.

##### Etymology.

The generic name is derived from “Myanmar” in combination with the *Orchestia* stem.

##### Diagnosis.

Body size medium. Eyes rounded or sub-rounded, approximately 1/3 of head length. Antenna I reaching mid-point of peduncular article V of antenna II, flagellum a little shorter than peduncle. Antenna II 28% of body length, flagellum a little longer than peduncle. Mandible left lacinia mobilis 4-dentate. Maxilliped palp with four articles, first two articles broad; article III not lobate distomedially, article IV distinct, short, with apical spine and setae.

Gnathopod I coxal plate with anterior process prominent, carpus lacking pellucid lobe, propodus simple, narrowed distally in both sexes. Gnathopod II sexually dimorphic; propodus of male transitional form, weakly chelate, with tumescence, propodus of female mitten-shaped. Pereopods simplidactylate, with two spines at hinge of unguis. Coxal gills present on gnathopod II and pereopods III–VI; each gill lobed and convoluted, one or two lobes with ridged margins or filamentous projections. Oostegites slender, present on gnathopod II and pereopods III–V.

Epimeral plates acuminate posterodistally, ventral margins without armature, lacking submarginal pits. Pleopods well-developed, peduncles with plumose setae on exterior margins. Uropod I peduncle with large distolateral spine, outer ramus marginally bare. Uropod III ramus shorter than peduncle. Telson subtriangular shaped, apically notched, each lobe with one or two apical spines.

##### Remarks.

*Myanmarorchestia* Hou, gen. n. is quite unique in having complex coxal gills which bears filamentous projections, simple gnathopod I in both sexes, and weakly chelate, transitional gnathopod II in male.

The new genus belongs to the *Orchestia* group. It is most similar to the genus *Bousfieldia* Chou & Lee, 1996 in left lacinia 4-dentate, well-developed pleopods and bare outer ramus in uropod I. It can be distinguished from *Bousfieldia* by the following characters (*Bousfieldia* in parentheses): gnathopod II in male subtriangle, with tumescence (strongly subchelate, oval in shape); pereopods simplidactylate (cuspidactylate); coxal gills of gnathopod II and pereopods III–VI lobed and convoluted, with ridged margins or filamentous projections (coxal gills lobed at middle, no filamentous projections).

The new genus can be distinguished from other terrestrial *Orchestia* groups from Indo-West Pacific margins (*Lanorchestia* Miyamoto & Morino, 2010, *Mizuhorchestia* Morino, 2014 and *Nipponorchestia* Morino & Miyamoto, 2015) by gnathopod II subtriangle (oval); pereopods simplidactylate (cuspidactylate); and pleopods well-developed (reduced).

This new genus shows morphological similarities to one landhopper genus from the Philippines, *Curiotalitrus* Lowry & Coleman, 2012 in sharing antenna I shorter than peduncular article V of antenna II; mandible left lacinia mobilis 4-dentate; article IV of maxilliped palp very short, well defined; uropod III ramus shorter than peduncle. It can be distinguished from *Curiotalitrus* by the following characters (*Curiotalitrus* in parentheses): gnathopod I carpus slightly expended near distal end, propodus stout (subchelate; posterior margins of carpus and propodus with palmate lobes); gnathopod II sexually dimorphic, propodus of male subtriangle, with tumescence (not sexually dimorphic, mitten-shaped); pleopods rami with numerous articles (fused 1-articulate); uropod I of male similar to that of the female (sexual dimorphism in uropod I); telson with 1–2 apical spines on each lobe (with 3–6 robust setae).

The new genus *Myanmarorchestia* is similar to landhopper genera *Arcitalitrus* Hurley, 1975, *Keratroides* Hurley, 1975 and *Mysticotalitrus* Hurley, 1975 in sharing following characters: left mandible lacinia mobilis 4-dentate; maxilliped palp article II unlobed, article IV distinct and small; gnathopod I simple; pereopods III–VII simplidactylate; and uropod I peduncle with distinct distolateral spine, outer ramus marginally bare. However, genera *Arcitalitrus*, *Keratroides* and *Mysticotalitrus* with more or less reduced pleopods, the peduncle lacking plumose setae; and telson not subtriangular in shape. *Solitroides* female is also similar to that of the new genus, but coxal gills not lobed, reduced pleopod III are distinguishing characters. The genus *Talitriator* Methuen, 1913 shares most features with the new genus, but gnathopod II in male is mitten-shaped and the telson is wider in middle. The peculiar gnathopod II in male of the new genus *Myanmarorchestia* is also exhibited in *Puhuruhuru patersoni* (Stephensen, 1938), *Parorchestia lesliensis* (Hurley, 1957), and *Platorchestia zachsi* (Derzhavin, 1937), but they can be distinguished from the new genus by cuspidactylate pereopods III–VII.

#### Myanmarorchestia peterjaegeri

Taxon classificationAnimaliaAmphipodaTalitridae

Hou sp. n.

http://zoobank.org/F02C69E9-261A-42B3-878F-C377EB462A67

**Figure 1.**
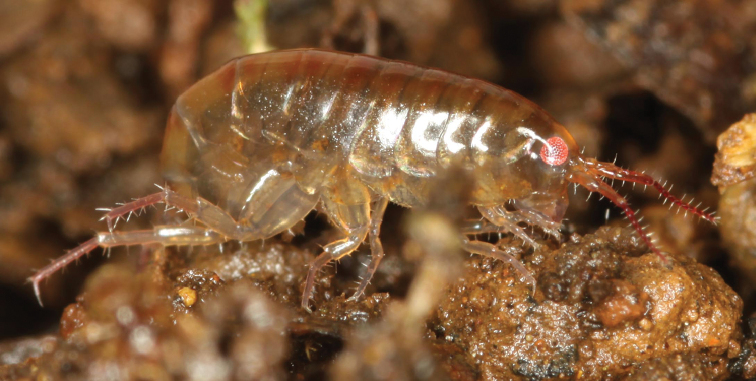
Life photo of *Myanmarorchestia peterjaegeri* Hou, sp. n., one of the female paratypes, photo courtesy of Peter Jäger.

**Figure 2.**
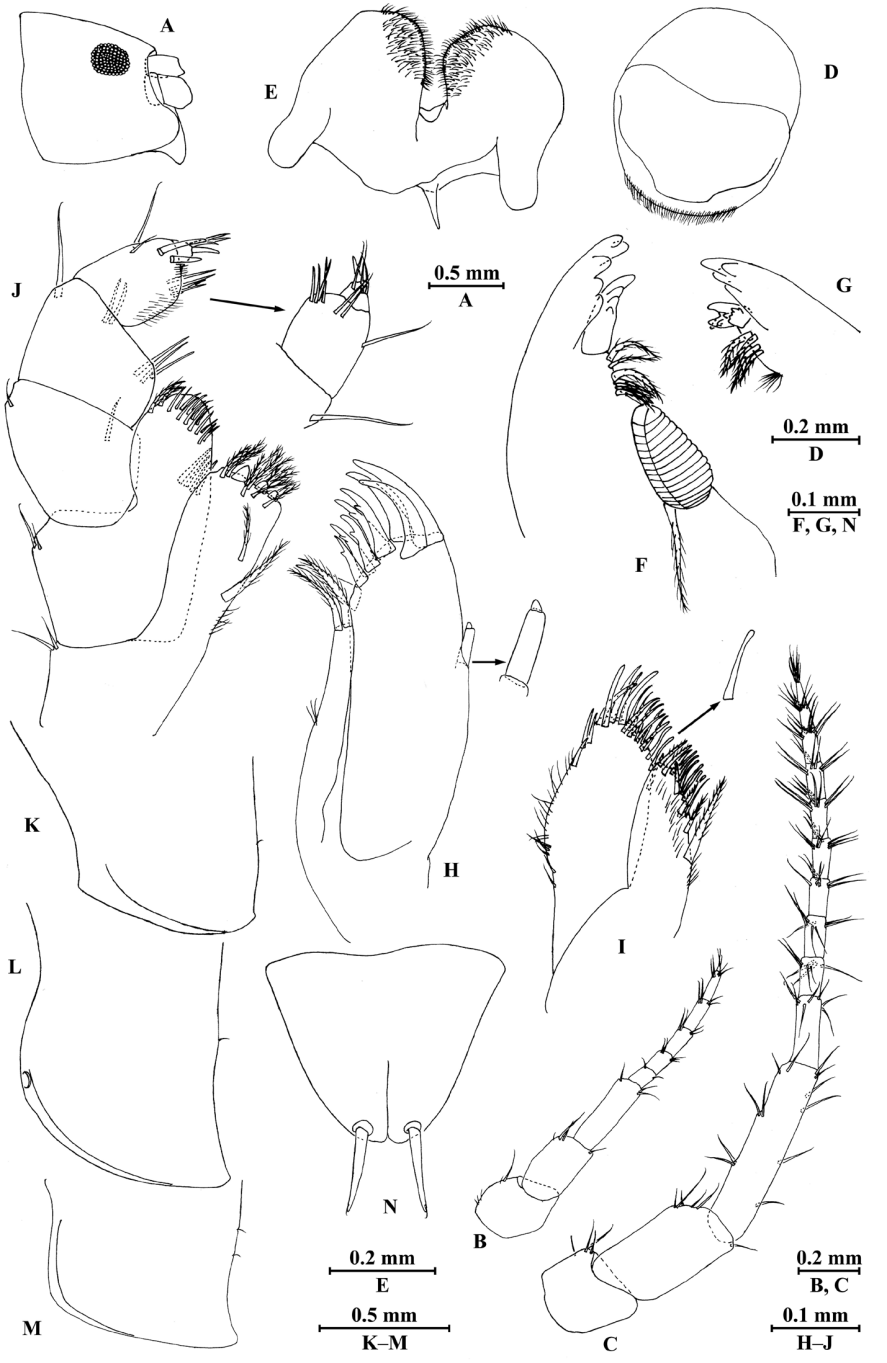
*Myanmarorchestia peterjaegeri* Hou, sp. n., female holotype. **A** head **B** antenna I **C** antenna II **D** upper lip **E** lower lip **F** left mandible **G** incisor of right mandible **H** left maxilla I **I** maxilla II **J** maxilliped **K** epimeral plate I **L** epimeral plate II **M** epimeral plate III **N** telson.

**Figure 3.**
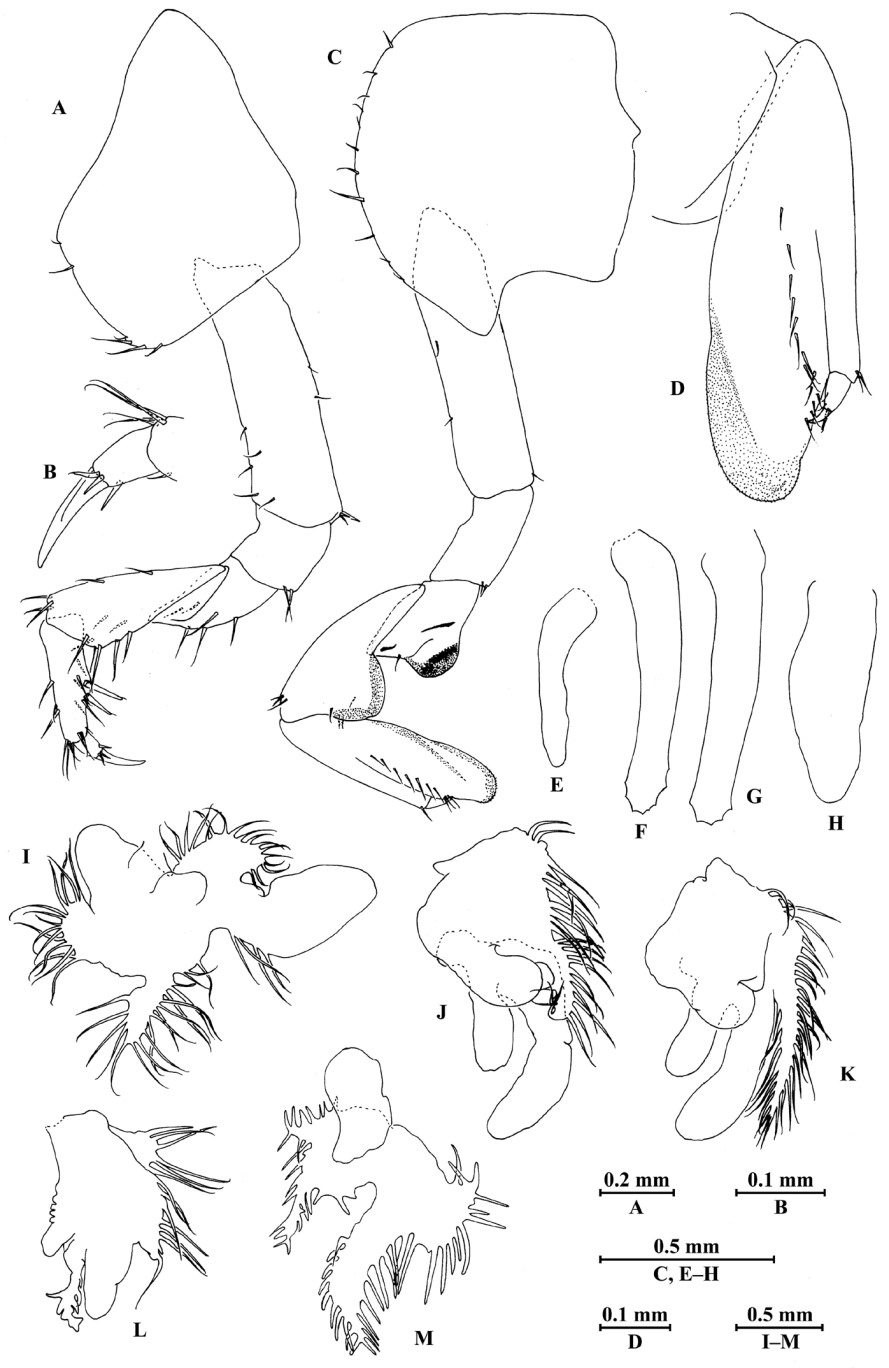
*Myanmarorchestia peterjaegeri* Hou, sp. n., female holotype. **A** gnathopod I **B** dactylus of gnathopod I **C** gnathopod II **D** propodus of gnathopod II **E** oostegite of gnathopod II **F** oostegite of pereopod III **G** oostegite of pereopod IV **H** oostegite of pereopod V **I** coxal gill of gnathopod II **J** coxal gill of pereopod III **K** coxal gill of pereopod IV **L** coxal gill of pereopod V **M** coxal gill of pereopod VI.

**Figure 4.**
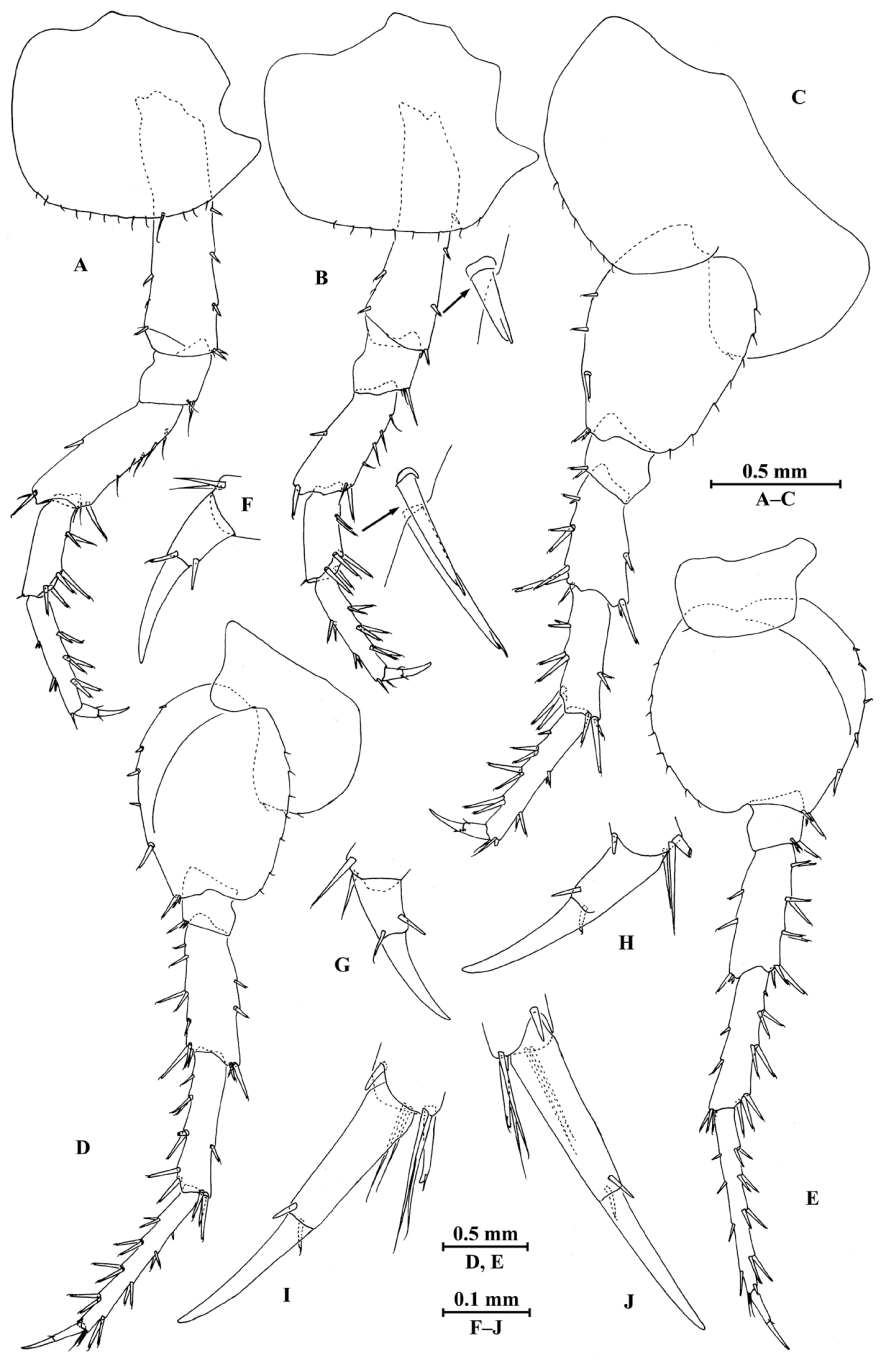
*Myanmarorchestia peterjaegeri* Hou, sp. n., female holotype. **A** pereopod III **B** pereopod IV **C** pereopod V **D** pereopod VI **E** pereopod VII (right) **F** dactylus of pereopod III **G** dactylus of pereopod IV **H** dactylus of pereopod V **I** dactylus of pereopod VI **J** dactylus of pereopod VII.

**Figure 5.**
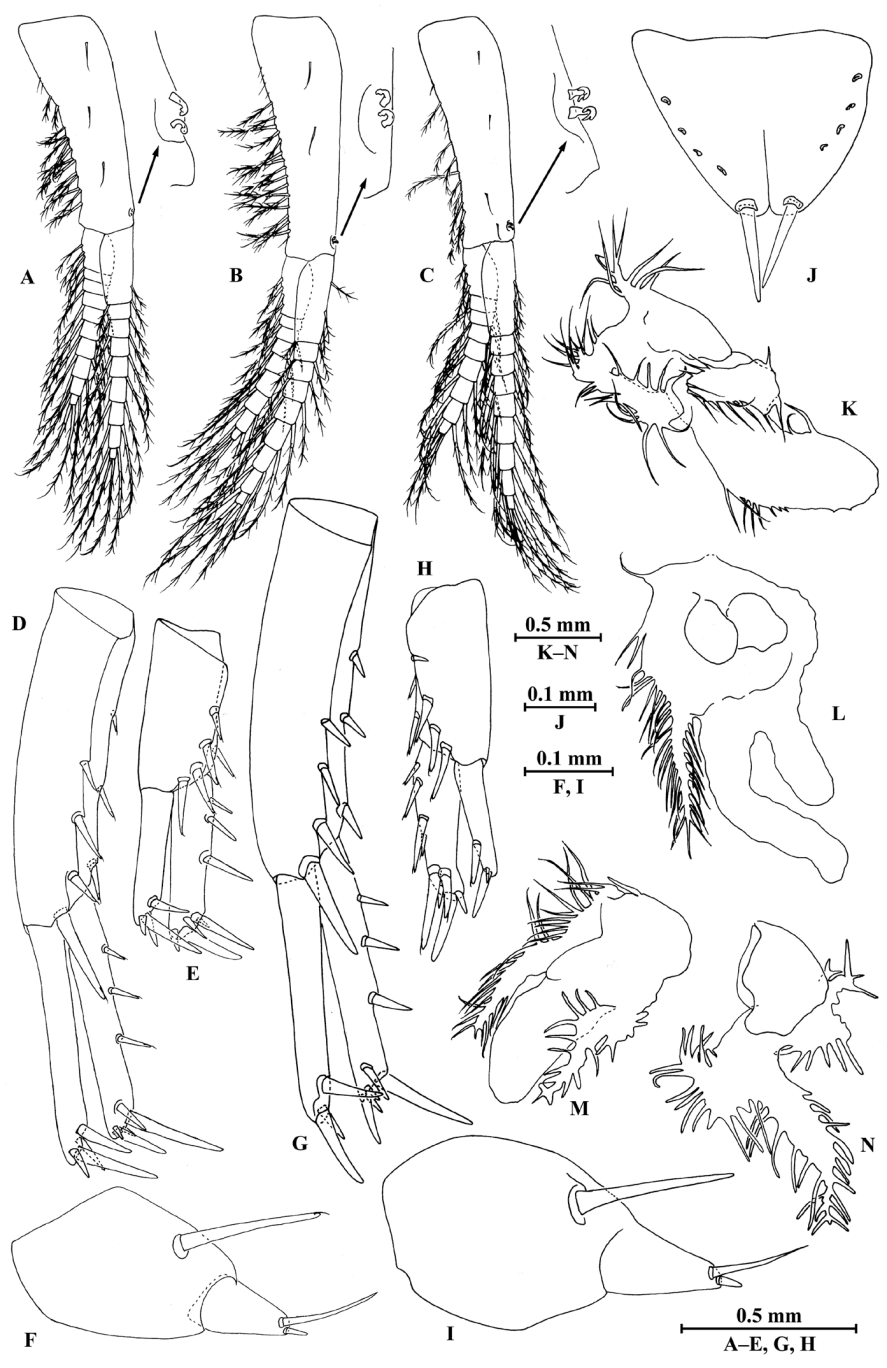
*Myanmarorchestia peterjaegeri* Hou, sp. n., **A–F** female, holotype; **G–N** male, paratype. **A** pleopod I **B** pleopod II **C** pleopod III **D** uropod I **E** uropod II **F** uropod III **G** uropod I **H** uropod II (right) **I** uropod III **J** telson **K** coxal gill of gnathopod II **L** coxal gill of pereopod III **M** coxal gill of pereopod V **N** coxal gill of pereopod VI.

**Figure 6.**
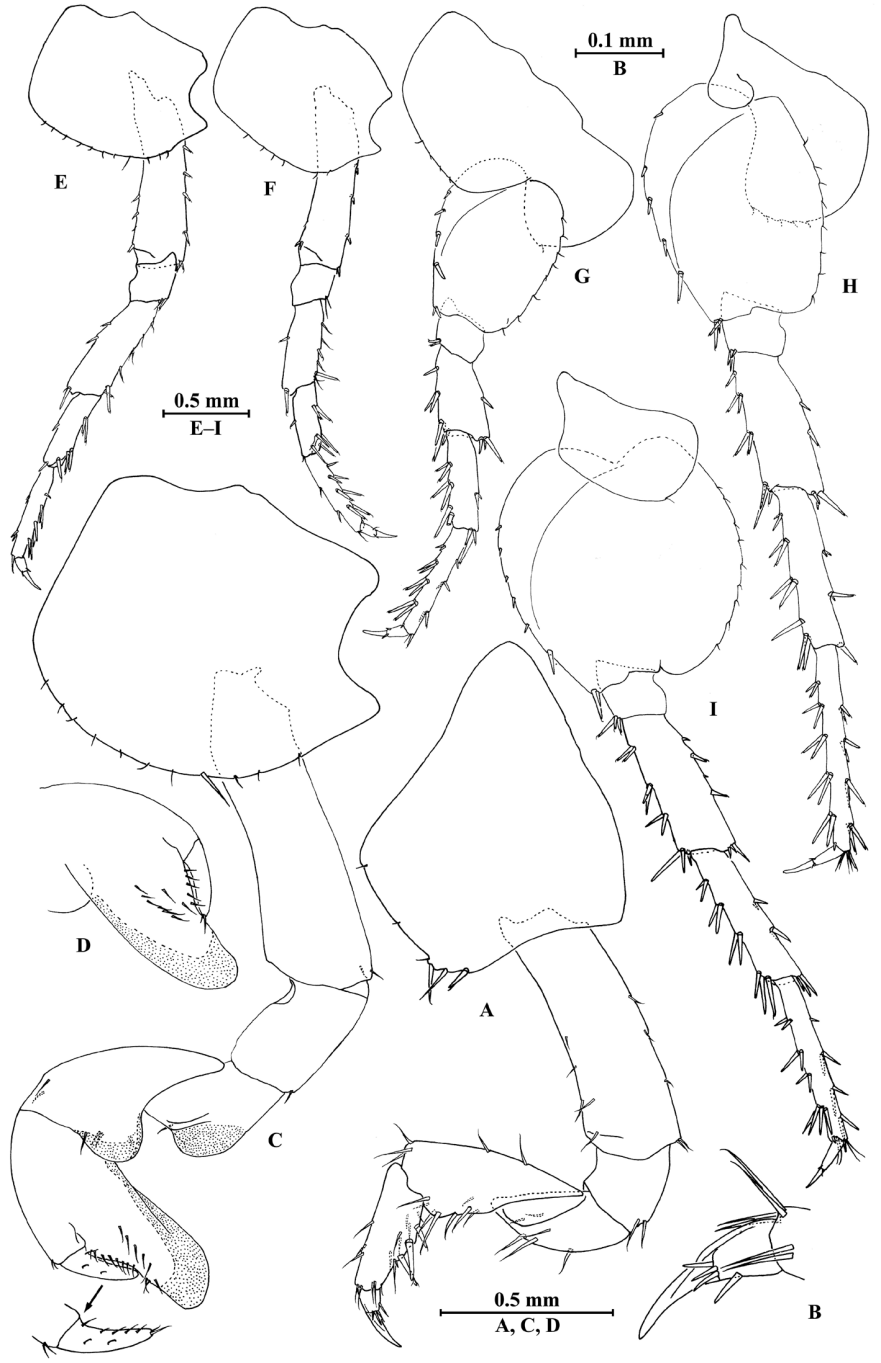
*Myanmarorchestia peterjaegeri* Hou, sp. n., male paratype. **A** gnathopod I **B** dactylus of gnathopod I **C** gnathopod II **D** propodus of gnathopod II **E** pereopod III **F** pereopod IV **G** pereopod V **H** pereopod VI **I** pereopod VII.

Figs 1 , 2 , 3 , 4 , 5 , 6 , 13

##### Material examined.

Holotype: female (IZCAS-I-A1692-1), 7.8 mm, Nat Ma Taung National Park (21.21°N, 94.02°E) (Mt. Victoria), altitude 2150 m, Chin State, Myanmar, May 11, 2014, collected by Peter Jäger. Paratype: male (SMF50714), 7.0 mm (without head), same data as holotype, GenBank accession number MF663279; paratypes, 3 females (SMF50715).

##### Etymology.

The specific name honours Peter Jäger, the collector of specimens used in this study and an excellent scientist supporting the diversity research in Myanmar; noun (name) in genitive case.

##### Diagnosis.

Eyes sub-rounded (Fig. 1); maxilla I palp with two articles, terminal article very small; gnathopod II merus and carpus protuberant on posterior margin; coxal gills convoluted, with marginal filamentous projections; telson with nicks on surface.

##### Description of holotype female

(IZCAS-I-A1692-1), 7.8 mm.

**Head.** (Fig. 2A): Eyes sub-rounded, medium in size, approx. 1/3 of head length.

*Antenna I* (Fig. 1; Fig. 2B): peduncle articles I–III in length ratio 1.0 : 1.2 : 1.4; flagellum with five articles and one fine distal article, a little shorter than peduncle, each article with short distal setae.

*Antenna II* (Fig. 2C): peduncle articles III–V in length ratio 1.0 : 1.7 : 2.3, with setae on anterior and posterior margins; flagellum with ten articles, each article with setae on dorsal and ventral margins.

*Upper lip* (Fig. 2D): ventral margin rounded, bearing minute setae.

*Mandible* (Fig. 2F, G): incisor of left mandible with five teeth; lacinia mobilis with four teeth; spine row with six plumose setae; molar with a plumose seta; incisor of right mandible with four teeth, lacinia mobilis bifurcate, with small teeth.

*Lower lip* (Fig. 2E): inner lobes indistinct, outer lobes covered with thin setae.

*Maxilla I* (Fig. 2H): inner plate with two terminal strong setae, outer plate with nine serrated apical spines, palp with two small articles.

*Maxilla II* (Fig. 2I): inner plate narrower and shorter than outer plate, with plumose and simple setae on medial margin, outer plate with two rows of apical spines.

*Maxilliped* (Fig. 2J): inner plate with three stout apical spines and numerous plumose setae; outer plate bearing four setae on interior margin, some simple setae and two plumose setae apically; palp with four articles, first two articles broad; articles I–III in length ratio 1.0 : 0.8 : 0.7; article II with three setae on interior side and one seta on exterior side; article III 0.6 times as wide as article II, with some setae and two spines, much wider than article IV; article IV short, with one spine and three simple setae apically.

**Pereon.** *Gnathopod I* (Fig. 3A, B): coxal plate bearing six setae on distal margin, anterior margin processed proximally; basis with fine setae on anterior and posterior margins; merus, carpus, and propodus in length ratio 1.0 : 1.8 : 1.2; merus bearing setae on posterior margin; carpus with setae on anterior and posterior margins; propodus with setae on anterior margin and two spines accompanied by setae on posterior margin; dactylus with one spine on posterior margin and two spines at hinge of unguis.

*Gnathopod II* (Fig. 3C, D): coxal plate with 11 setae on distal margin, posterior process prominent; basis with two fine setae on anterior margin and one seta on posterior margin; merus protuberant on posterior margin; carpus 1.5 times as long as wide, with tumescent hump at posterodistal corner, and several fine setae at base of hump, anterior margin with three setae; propodus with tumescence, with setae on surface and palm margin; dactylus with two setae on posterior margin and one seta at hinge of unguis.

*Pereopod III* (Fig. 4A, F): coxal plate with posterior cusp, bearing 13 setae on distal margin; basis longest, with spines accompanied by fine setae on anterior and posterior margins; merus, carpus, and propodus in length ratio 1.0 : 0.7 : 0.9; carpus and propodus with spines on posterior margins; dactylus not pinched, with two spines at hinge of unguis. Pereopods III–VII simplidactylate.

*Pereopod IV* (Fig. 4B, G): similar to pereopod III; slightly shorter; coxal plate with posterior cusp, bearing eight setae on ventral margin; merus, carpus, and propodus in length ratio 1.0 : 0.8 : 1.1; dactylus not pinched.

*Pereopod V* (Fig. 4C, H): coxal plate bilobed, anterior lobe bigger than posterior lobe, bearing six setae and one seta on anterior and posterior lobes, respectively; basis suboval, with three spines on anterior margin and seven setae on posterior margin, anterodistal corner with two spines; merus, carpus, and propodus in length ratio 1.0 : 1.0 : 1.2, with spines on both margins; dactylus with two spines at hinge of unguis.

*Pereopod VI* (Fig. 4D, I): coxal plate bilobed, anterior lobe much smaller than posterior lobe, bearing three setae on posterior lobe; basis suboval, with five spines on anterior margin and seven setae on posterior margin, anterodistal corner with three spines; merus, carpus, and propodus in length ratio 1.0 : 1.0 : 1.3, with spines on both margins; propodus and dactylus slender, dactylus with two spines at hinge of unguis.

*Pereopod VII* (Fig. 4E, J): coxal plate non-lobate, shallow, posterodistal corner with one seta; basis oval, with five spines on anterior margin and eight setae on posterior margin, anterodistal corner with three spines; merus, carpus, and propodus in length ratio 1.0 : 1.1 : 1.5, with spines on both margins; propodus and dactylus slender, dactylus with two spines at hinge of unguis.

*Coxal gills* (Fig. 3I–M): present on gnathopod II and pereopods III–VI; gill of gnathopod II lobed, each lobe with marginal filamentous projections; gill of pereopods III and IV similar, lobed and convoluted, one lobe with marginal filamentous projections; gill of pereopod V smallest, lobed and convoluted, one lobe with marginal filamentous projections and one lobe ridged; gill of pereopod VI lobed and convoluted, each lobe ridged.

*Oostegites* (Fig. 3E–H): slender, present on gnathopod II and pereopods III–V.

**Pleon.** *Epimeral plates* (Fig. 2K–M): acuminate posterodistally, distal margins without armature; plate I with one fine seta on posterior margin; plate II with one fine seta on posterior margin, anterodistal corner with one nick; plate III shorter, with two fine setae on posterior margin.

*Pleopods I–III* (Fig. 5A–C): similar, peduncle with two retinacula on interior margin, exterior margin with dense plumose setae and fine setae; outer ramus 85% of peduncle, outer ramus shorter than inner ramus, both inner and outer rami fringed with plumose setae.

**Urosome.** *Uropods I–III* (Fig. 5D–F): uropod I peduncle longer than rami, with three spines on interior margin and four spines on exterior margin, distolateral spine approx. twice of subdistal one; inner ramus with three spines on interior side and five terminal spines; outer ramus with four terminal spines. Uropod II short, peduncle bearing one spine on interior margin and four spines on exterior margin; inner ramus with three spines on interior side and five terminal spines; outer ramus slightly shorter than inner ramus, with four terminal spines. Uropod III peduncle expanded, with one long posterodistal spine; ramus short, 0.5 times as long as peduncle, with one long slender spine and one short spine apically.

*Telson* (Fig. 2N): notched, with suture on distal 1/3, each lobe with one apical spine.

##### Description of paratype male

(SMF50714), head missing, 7.0 mm.

**Pereon.** *Gnathopod I* (Fig. 6A, B): similar to that of female, coxal plate bearing five setae and one spine on distal margin, anterior margin processed proximally; basis with fine setae on anterior and posterior margins; merus, carpus, and propodus in length ratio 1.0 : 1.4 : 1.0; merus bearing setae on posterior margin; carpus with setae on anterior and posterior margins; propodus with setae on anterior margin and three spines accompanied by setae on posterior margin; dactylus with one spine on posterior margin and three spines at hinge of unguis.

*Gnathopod II* (Fig. 6C, D): coxal plate with 11 setae on distal margin, posterior process prominent; basis slender, anterior margin weakly sinuate, with two fine setae on posterodistal corner; merus protuberant on posterior margin; carpus with tumescent hump at posterodistal corner, and several fine setae at base of hump, anterior margin with two setae; propodus anteroventrally produced, with tumescence, wider than that of female, with setae on surface and palm margin; dactylus robust, with two nicks at surface, five setae on posterior margin and two setae at tip.

*Pereopods III–VII* (Fig. 6E–I): similar to those of female.

*Coxal gills* (Fig. 5K–N): similar to those of female, coxal gill of pereopod IV lost.

**Urosome.** As is the female except: Uropod I (Fig. 5G) outer ramus with three terminal spines; Uropod II (Fig. 5H) peduncle bearing two spines on interior margin and four spines on exterior margin, inner ramus with four spines on interior side and five terminal spines; outer ramus with three terminal spines; Uropod III (Fig. 5I) peduncle a little wider than that of female.

*Telson* (Fig. 5J): notched, each lobe with four nicks on surface and one apical spine.

##### Habitat.

This species was collected from a disturbed primary forest of the Nat Ma Taung National Park (Fig. 13).

##### Remarks.

The new species is characterised by the complex lobed gills with filamentous projections. This structure may be related to its terrestrial habitats at high elevations. The convoluted feature can increase the surface area of the gills to keep respiration.

#### Myanmarorchestia seabri

Taxon classificationAnimaliaAmphipodaTalitridae

Hou sp. n.

http://zoobank.org/5BE7DB41-C778-4EE0-961F-75FA4CD3DD8C

**Figure 7.**
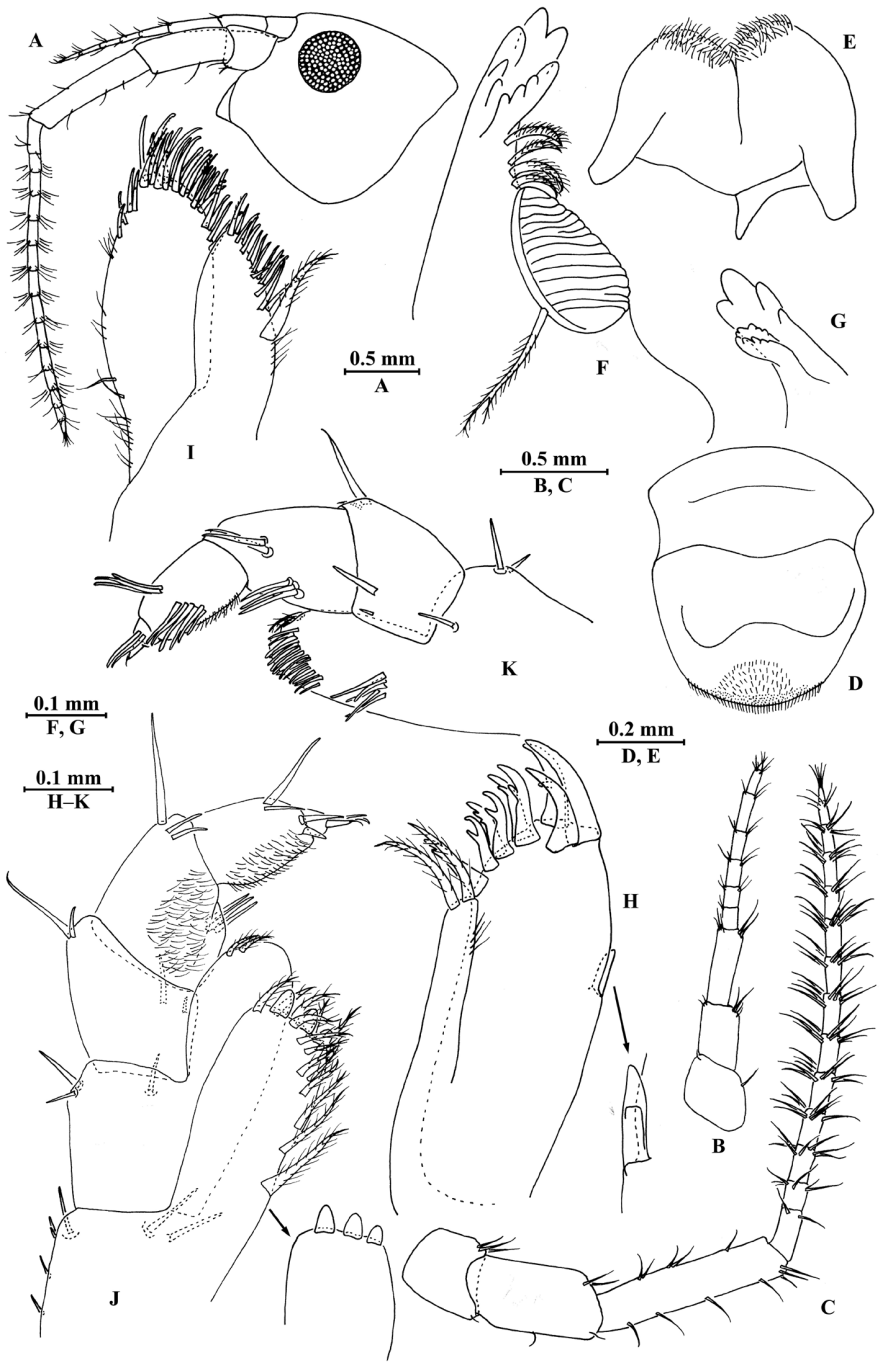
*Myanmarorchestia seabri* Hou, sp. n., male holotype. **A** head **B** antenna I **C** antenna II **D** upper lip **E** lower lip **F** left mandible **G** incisor of right mandible **H** left maxilla I **I** maxilla II **J** maxilliped **K** outer plate and palp of left maxilliped.

**Figure 8.**
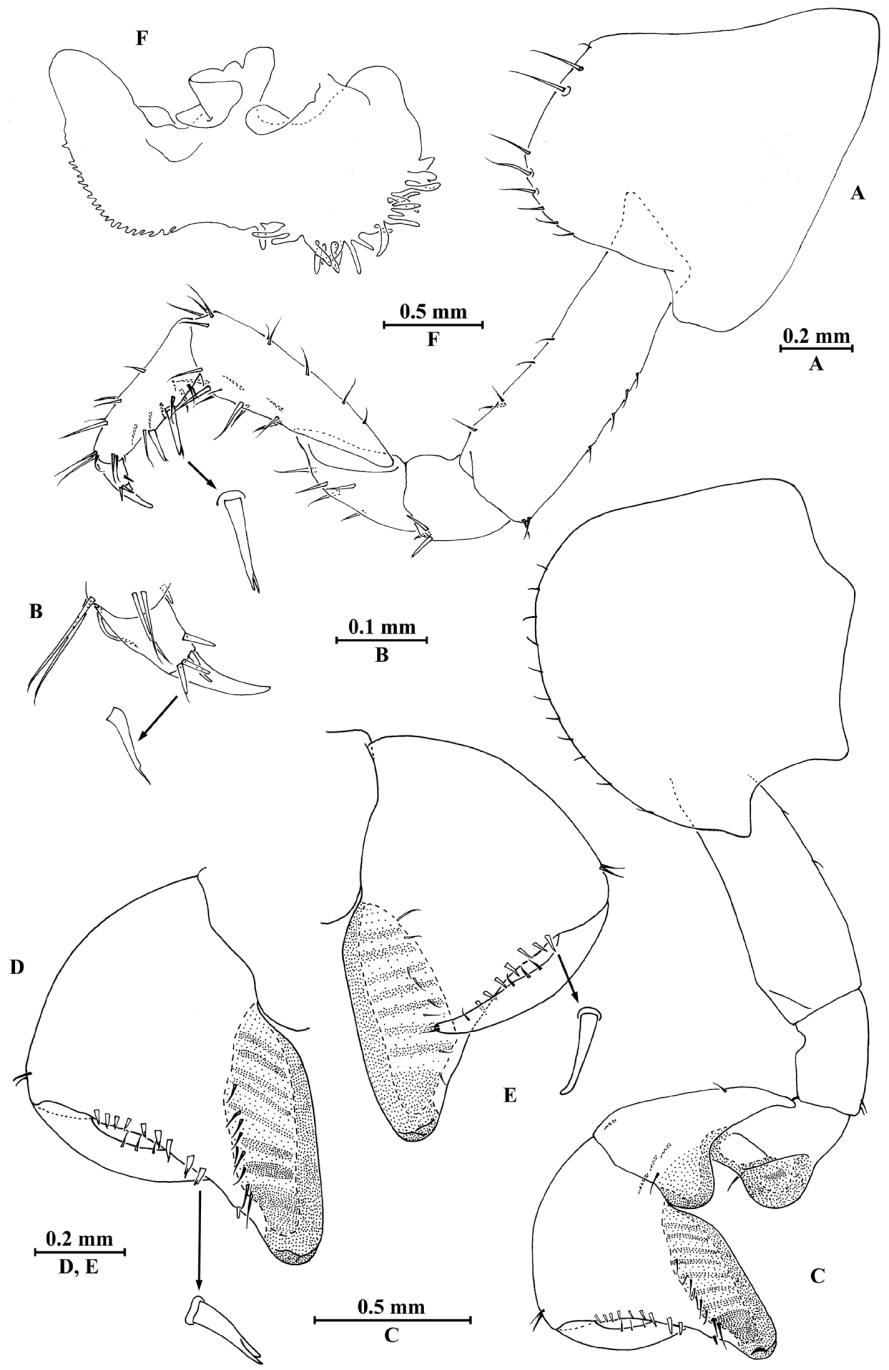
*Myanmarorchestia seabri* Hou, sp. n., male holotype. **A** gnathopod I **B** dactylus of gnathopod I **C** gnathopod II **D** propodus of gnathopod II **E** propodus of gnathopod II (dorsal view) **F** coxal gill of gnathopod II.

**Figure 9.**
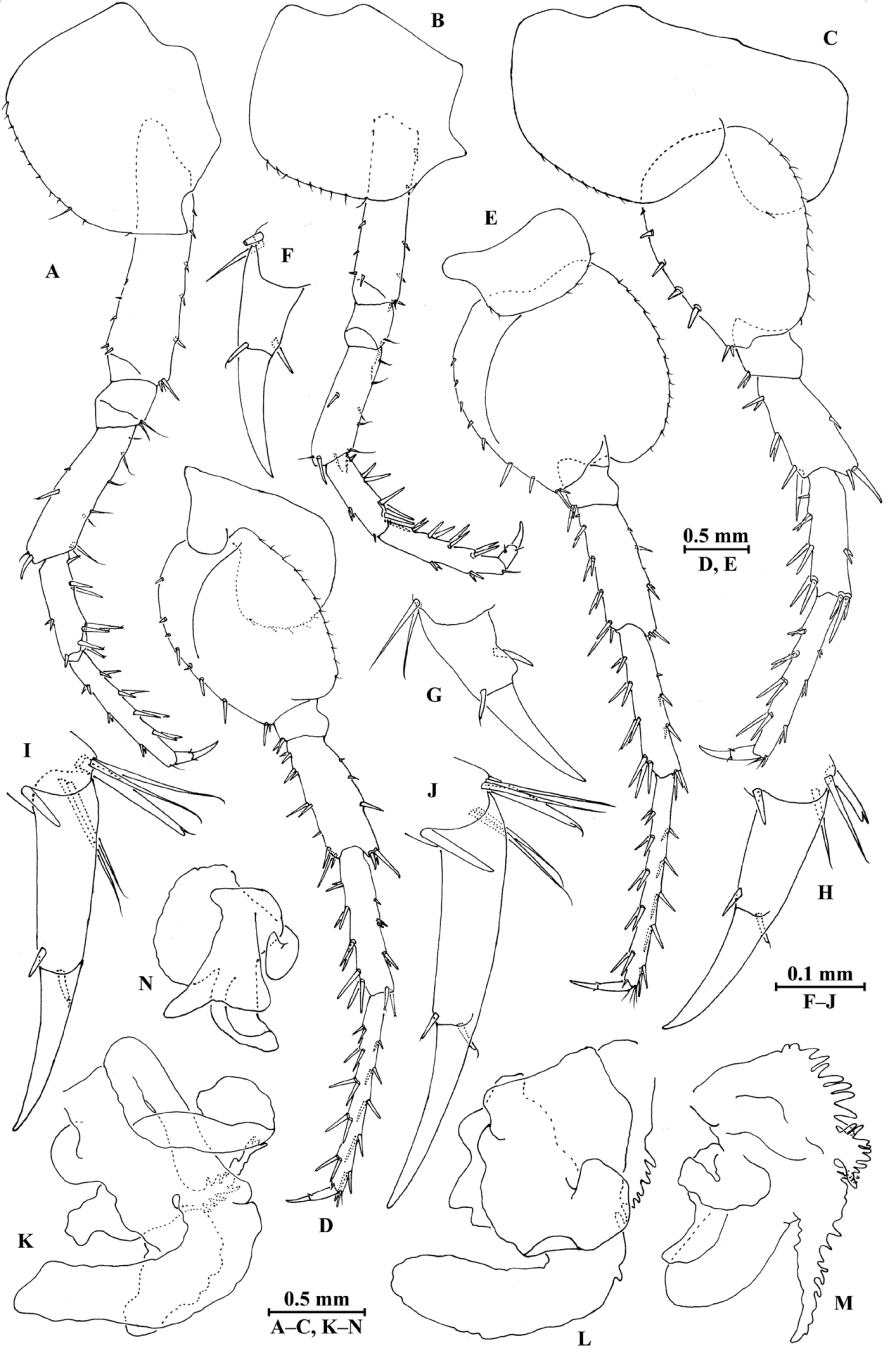
*Myanmarorchestia seabri* Hou, sp. n., male holotype. **A** pereopod III **B** pereopod IV **C** pereopod V **D** pereopod VI **E** pereopod VII **F** dactylus of pereopod III **G** dactylus of pereopod IV **H** dactylus of pereopod V **I** dactylus of pereopod VI **J** dactylus of pereopod VII **K** coxal gill of pereopod III **L** coxal gill of pereopod IV **M** coxal gill of pereopod V **N** coxal gill of pereopod VI.

**Figure 10.**
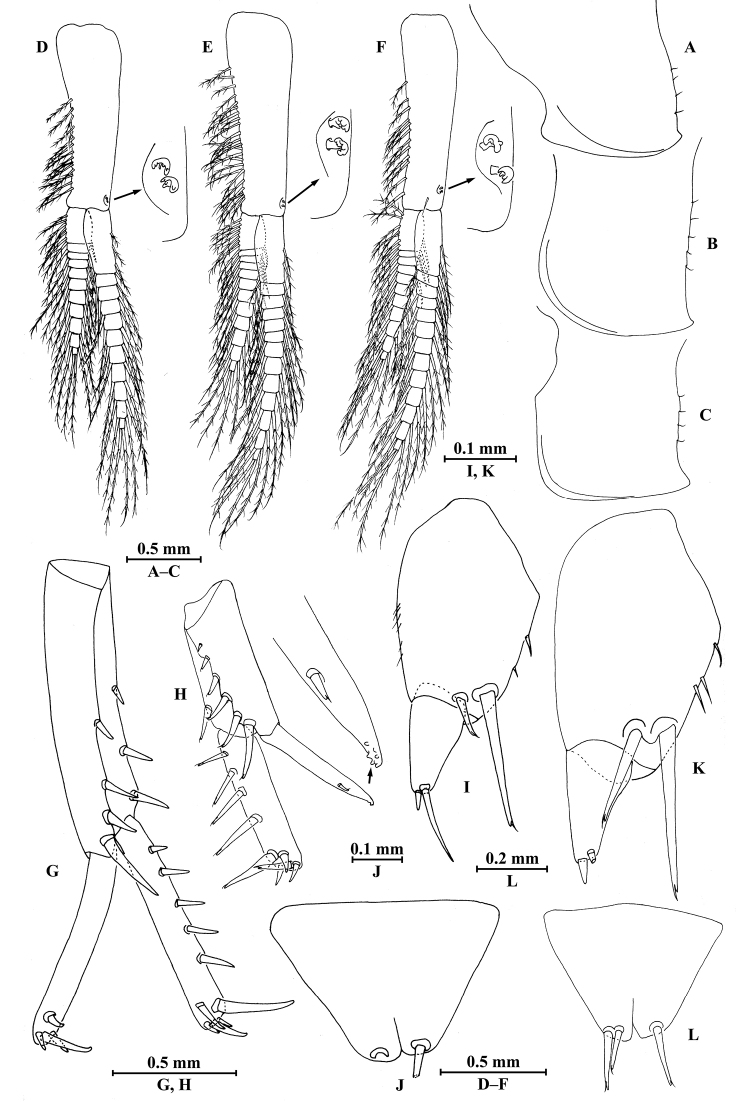
*Myanmarorchestia seabri* Hou, sp. n., **A–J** male, holotype; **K, L** female, paratype. **A** epimeral plate I **B** epimeral plate II **C** epimeral plate III **D** pleopod I **E** pleopod II **F** pleopod III **G** uropod I **H** uropod II (right) **I** uropod III **J** telson **K** uropod III **L** telson.

**Figure 11.**
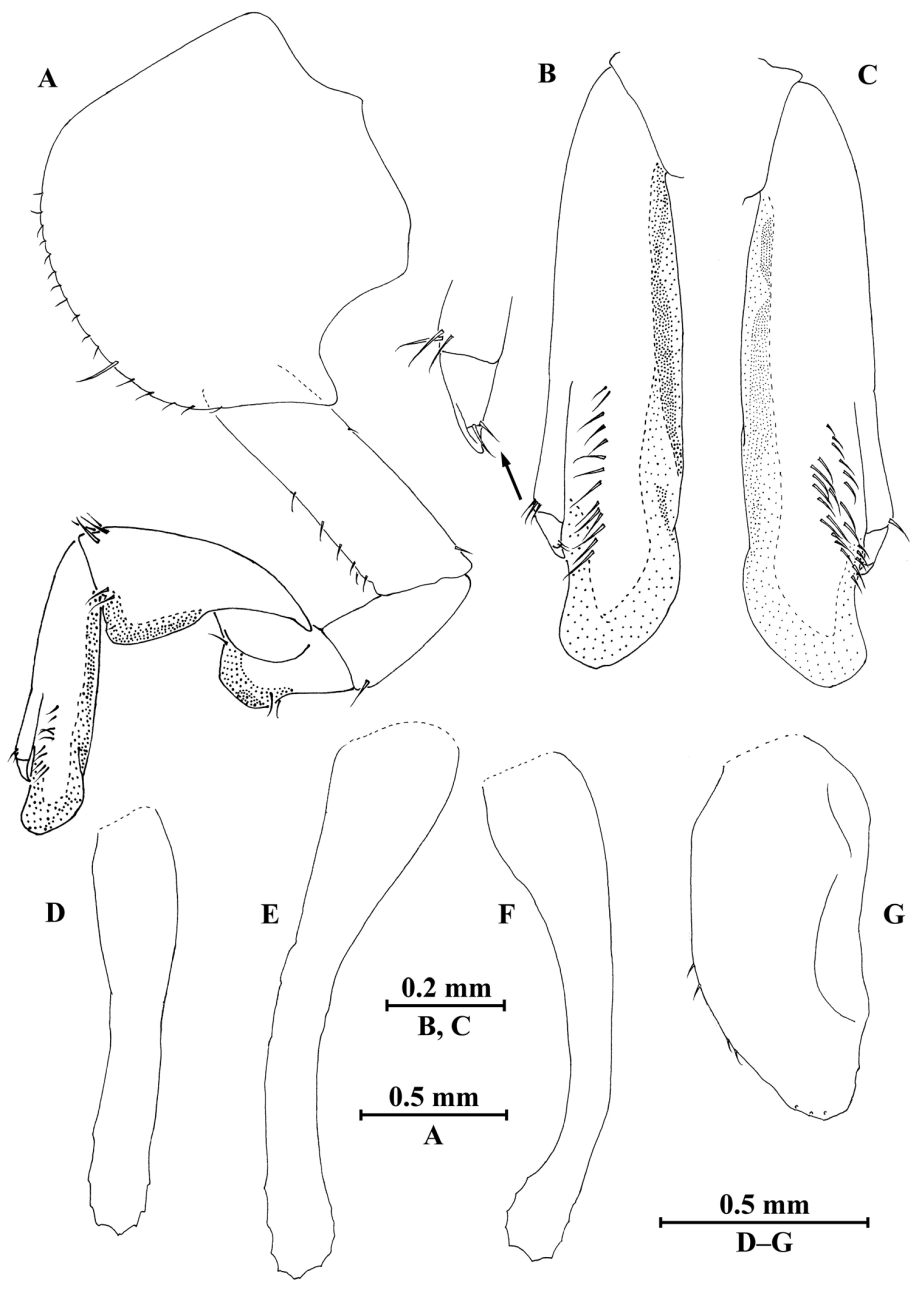
*Myanmarorchestia seabri* Hou, sp. n., female paratype. **A** gnathopod II **B** dactylus of gnathopod II **C** dactylus of gnathopod II (dorsal view) **D** oostegite of gnathopod II **E** oostegite of pereopod III **F** oostegite of pereopod IV **G** oostegite of pereopod V.

**Figure 12.**
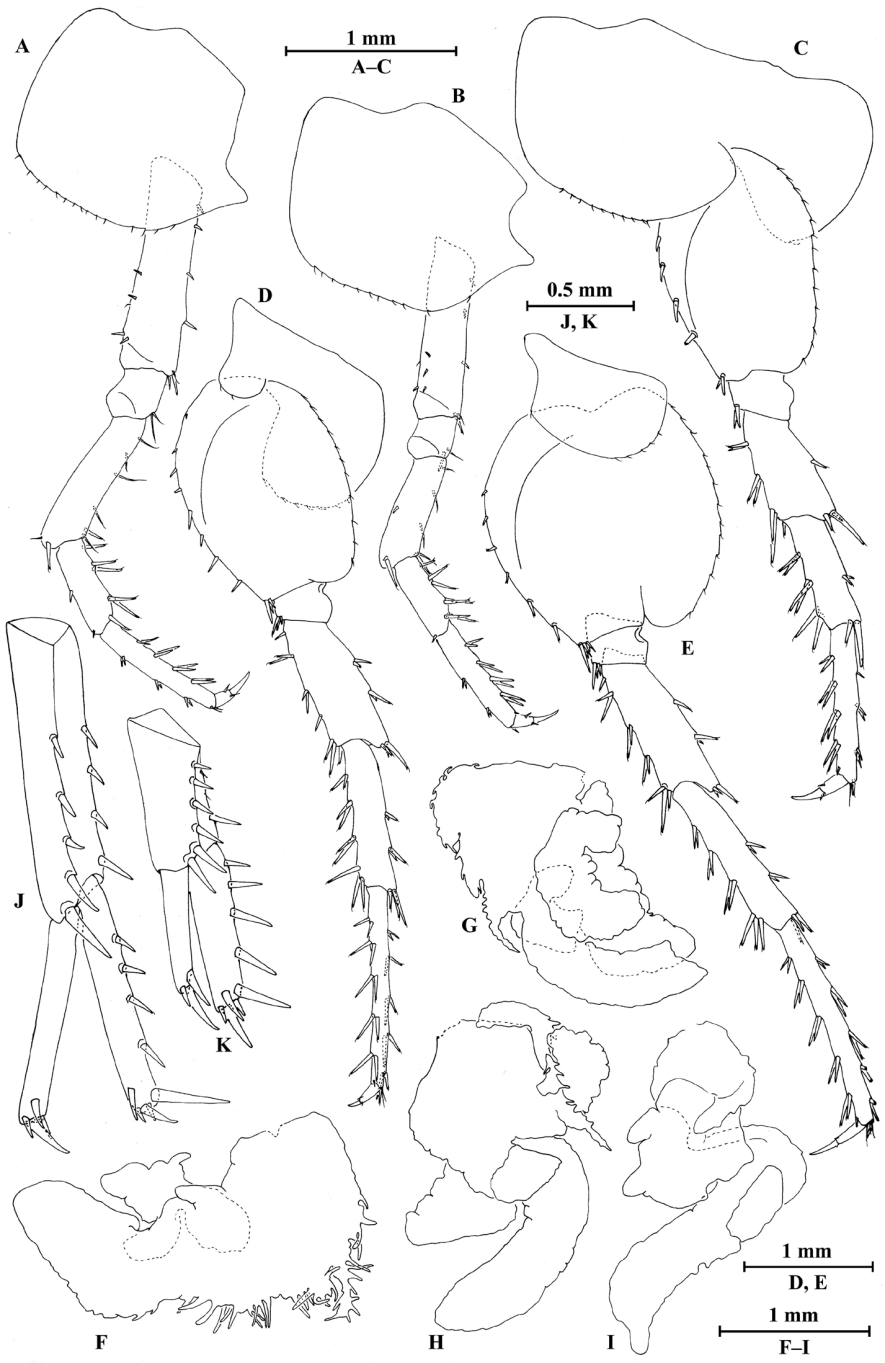
*Myanmarorchestia seabri* Hou, sp. n., female paratype. **A** pereopod III **B** pereopod IV **C** pereopod V **D** pereopod VI **E** pereopod VII **F** coxal gill of gnathopod II **G** coxal gill of pereopod III **H** coxal gill of pereopod IV **I** coxal gill of pereopod VI **J** uropod I **K** uropod II.

**Figure 13.**
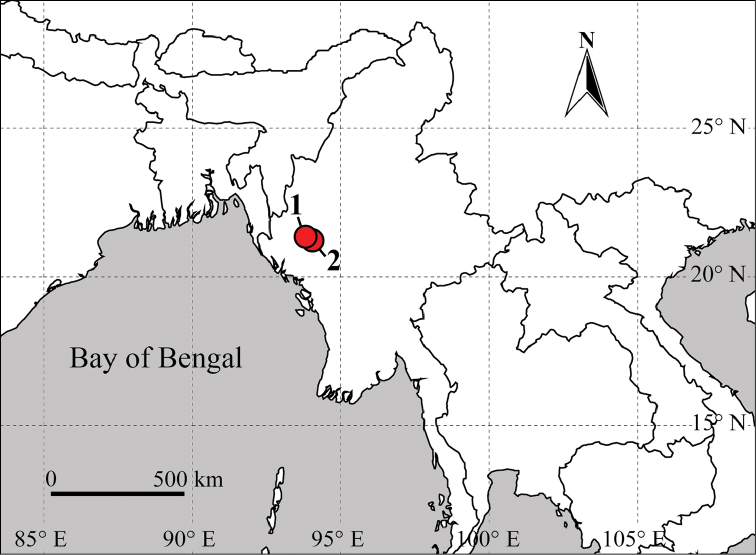
Collection localities of *Myanmarorchestia* species from Myanmar. **1** *Myanmarorchestia peterjaegeri* Hou, sp. n. **2** *M. seabri* Hou, sp. n.

Figs 7 , 8 , 9 , 10 , 11 , 12 , 13

##### Material examined.

Holotype: male (IZCAS-I-A1690-1), 10.0 mm, Kampetlet (21.20°N, 94.03°E), altitude 1585 m, Chin State, Myanmar, May 17, 2014, collected by Peter Jäger. Paratype: female (SMF50716), 10.0 mm (head and gnathopod I missing), same data as holotype, GenBank accession number MF663278; paratypes, 2 males (SMF50717).

##### Etymology.

The specific name is derived from abbreviation of the Southeast Asia Biodiversity Research Institute, Chinese Academy of Sciences (CAS-SEABRI); noun in apposition.

##### Diagnosis.

Eyes rounded; maxilla I palp with one article; male gnathopod II merus and carpus protuberant on posterior margin, propodus with tumescence, sub-triangular; coxal gills convoluted; telson bare on surface.

##### Description of male.

Holotype (IZCAS-I-A1690-1), 10.0 mm.

**Head.** (Fig. 7A): Eyes rounded, medium in size.

*Antenna I* (Fig. 7B): peduncle articles I–III in length ratio 1.0 : 1.1 : 1.4; flagellum with six articles and one fine distal article, a little shorter than peduncle, each article with short distal setae.

*Antenna II* (Fig. 7C): peduncle articles III–V in length ratio 1.0 : 2.0 : 2.8, with setae on anterior and posterior margins; flagellum with 14 articles, each article with setae on dorsal and ventral margins.

*Upper lip* (Fig. 7D): ventral margin rounded, bearing minute setae.

*Mandible* (Fig. 7F, G): incisor of left mandible with five teeth; lacinia mobilis with four teeth; spine row with four plumose setae; molar with a plumose seta; incisor of right mandible with four teeth, lacinia mobilis bifurcate, with small teeth.

*Lower lip* (Fig. 7E): inner lobes indistinct, outer lobes covered with thin setae.

*Maxilla I* (Fig. 7H): inner plate with two terminal strong setae, outer plate with nine serrated apical spines, palp with one fused article.

*Maxilla II* (Fig. 7I): inner plate narrower and shorter than outer plate, with one plumose seta and numerous simple setae on medial margin, outer plate with two rows of apical spines.

*Maxilliped* (Fig. 7J, K): inner plate with three stout apical spines and 14 plumose setae; outer plate bearing five setae on interior margin, some simple setae and two plumose setae apically; palp with four articles, first two articles broad; articles I–III in length ratio 1.0 : 1.0 : 0.6; articles II-III with fine setae; article III with two spines on distal end, two setae on exterior margin and five setae on ventral surface; article IV very short, with one spine and two simple setae apically.

**Pereon.** *Gnathopod I* (Fig. 8A, B): coxal plate bearing nine setae on distal margin, anterior margin processed proximally; basis with fine setae on anterior and posterior margins; merus, carpus, and propodus in length ratio 1.0 : 1.7 : 1.1; merus bearing setae on posterior margin; carpus with setae on anterior and posterior margins; propodus with setae on anterior margin and two spines accompanied by setae on posterior margin; dactylus with one spine on posterior margin and three spines at hinge of unguis.

*Gnathopod II* (Fig. 8C–E): coxal plate distal margin with eleven setae, posterior process prominent; basis with two fine setae on posterior margin; merus protuberant on posterior margin; carpus 1.4 times as long as wide, with tumescent hump at posterodistal comer; propodus with tumescence, subtriangle, with setae on surface, palm margin with a row of spines on interior and exterior sides; dactylus as long as palm, with setae on posterior margin and two setae at tip.

*Pereopod III* (Fig. 9A, F): coxal plate with posterior cusp, bearing 12 setae on distal margin; basis longest, with spines on anterior and posterior margins; merus, carpus, and propodus in length ratio 1.0 : 0.7 : 1.0; carpus and propodus with spines on posterior margins; dactylus with two spines at hinge of unguis. Pereopods III–IV simplidactylate.

*Pereopod IV* (Fig. 9B, G): similar to pereopod III; shorter; coxal plate with posterior cusp, bearing ten setae on ventral margin; merus, carpus, and propodus in length ratio 1.0 : 0.8 : 1.1, dactylus weakly pinched.

*Pereopod V* (Fig. 9C, H): coxal plate bilobed, anterior lobe bigger than posterior lobe, bearing seven setae and one seta on anterior and posterior lobes, respectively; basis suboval, with five spines on anterior margin and 11 setae on posterior margin, anterodistal corner with two spines; merus, carpus, and propodus in length ratio 1.0 : 1.2 : 1.7, with spines on both margins; dactylus with two spines at hinge of unguis.

*Pereopod VI* (Fig. 9D, I): coxal plate bilobed, anterior lobe much smaller than posterior lobe, bearing five setae on posterior lobe; basis suboval, with seven spines on anterior margin and 12 setae on posterior margin, anterodistal corner with two spines; merus, carpus, and propodus in length ratio 1.0 : 1.2 : 1.6, with spines on both margins; propodus and dactylus slender, dactylus with two spines at hinge of unguis.

*Pereopod VII* (Fig. 9E, J): coxal plate non-lobate, shallow, with two setae on anterodistal corner and four setae on posterodistal; basis oval, with seven spines on anterior margin and 14 setae on posterior margin, anterodistal corner with two spines; merus, carpus, and propodus in length ratio 1.0 : 1.2 : 1.7, with spines on both margins; propodus and dactylus slender, dactylus with two spines at hinge of unguis.

*Coxal gills* (Figs 8F, 9K–N): present on gnathopod II and pereopods III–VI; gill of gnathopod II and pereopods III–V lobed and convoluted, one or two lobes with ridged margins; gill of pereopod VI lobed and convoluted.

**Pleon.** *Epimeral plates* (Fig. 10A–C): acuminate posterodistally, distal margins without armature; plate I with four fine setae on posterior margin; plate II with five fine setae on posterior margin; plate III shorter, with four fine setae on posterior margin.

*Pleopods I–III* (Fig. 10D–F): similar, peduncle with two retinacula on interior margin, exterior margin with dense plumose setae; outer ramus approx. 80% of peduncle, outer ramus shorter than inner ramus, both inner and outer rami fringed with plumose setae.

**Urosome.** *Uropods I–III* (Fig. 10G–I): uropod I peduncle longer than rami, with three spines on interior margin and four spines on exterior margin; inner ramus with five spines on interior side and five terminal spines; outer ramus with four terminal spines. Uropod II short, peduncle bearing one spine on interior margin and six spines on exterior margin; inner ramus with four spines on interior side and five terminal spines; outer ramus slightly shorter than inner ramus, variation in armature, the holotype and one paratype with one spine on interior side and some small teeth distally, while the other paratype marginally bare but with three terminal spines as that of female (Fig. 12K). Uropod III peduncle expanded, with fine setae on ventral margin, two robust setae on dorsal margin and two posterodistal spines; ramus short, 0.6 times as long as peduncle, with one long slender spine and one short spine apically.

*Telson* (Fig. 10J): notched, each lobe with one apical spine.

##### Description of paratype female

(SMF50716), head and gnathopod I missing, 10.0 mm.

**Pereon.** *Gnathopod II* (Fig. 11A–C): coxal plate distal margin with 17 setae, posterior process prominent; basis slender, with six fine setae on anterior margin and two fine setae on posterior margin; merus protuberant on posterior margin; carpus with tumescent hump at posterodistal comer, with two setae on distal end and four setae on anterior margin; propodus with tumescence, with setae on surface and palm margin; dactylus with two setae at tip.

*Pereopods III–VII* (Fig. 12A–E): similar to those of male.

*Coxal gills* (Fig. 12F–I): similar to those of male, coxal gill of pereopod V lost.

*Oostegites* (Fig. 11D–G): present on gnathopod II and pereopods III–V, oostegites of gnathopod II and pereopods III–IV slender, oostegites of pereopod V expanded and smallest.

**Urosome.** *Uropods I–III* (Figs 12J, K; 10K): uropod I (Fig. 12J) peduncle longer than rami, with four spines on interior margin and five spines on exterior margin; inner ramus with four spines on interior side and five terminal spines; outer ramus with four terminal spines. Uropod II (Fig. 12K) short, peduncle bearing two spines and one fine seta on interior margin and six spines on exterior margin; inner ramus with four spines on interior side and five terminal spines; outer ramus shorter than inner ramus, with three terminal spines. Uropod III (Fig. 10K) peduncle expanded, with three stiff setae on dorsal margin and two posterodistal spines; ramus short, 0.5 times as long as peduncle, with one long slender spine and one short spine apically.

*Telson* (Fig. 10L): notched, left lobe with two apical spines and right lobe with one apical spine.

##### Habitat.

This species was collected by sieving leaf litter along a stream in a secondary forest (Fig. 13).

##### Remarks.

*Myanmarorchestia seabri* Hou, sp. n. can be distinguished from *M. peterjaegeri* Hou, sp. n. by the following characters (*M. peterjaegeri* Hou, sp. n. in parentheses): palp of maxilla I with one fused article (with two small articles); gnathopod II of male subtriangle, with bigger tumescence than that of *M. peterjaegeri* Hou, sp. n., palm margin with a row of spines on interior and exterior sides (with setae), dactylus elongate (short); coxal gills with one or two lobes ridged (with more filamentous projections or ridged margins); uropod III peduncle with two posterodistal spines (with one posterodistal spine); telson surface bare (with nicks). In addition, *M. peterjaegeri* Hou, sp. n. and *M. seabri* inhabit separate habitats, with up to 500 m elevation difference.

The uncorrected *p*-distance between *M. peterjaegeri* Hou, sp. n. and *M. seabri* Hou, sp. n. is 18.8% for COI. This significant differentiation confirmed that they are two different new species, in comparison with previous molecular threshold used for crustacean species delimitation (Lefébure et al. 2006, Hou et al. 2009).

## Supplementary Material

XML Treatment for Myanmarorchestia

XML Treatment for Myanmarorchestia peterjaegeri

XML Treatment for Myanmarorchestia seabri
